# An observational mixed-methods approach to investigate the fear of cancer recurrence cognitive and emotional model by Lee-Jones *et al* with women with breast cancer during radiotherapy treatment

**DOI:** 10.3332/ecancer.2019.984

**Published:** 2019-12-12

**Authors:** Isabel Del Mar Hita Millan, Josie Cameron, Yuan Yang, Gerry Humphris

**Affiliations:** 1 Medical School, University of St Andrews, St Andrews KY16 9AJ, UK; 2 Edinburgh Cancer Centre, Western General Hospital, Edinburgh EH4 2XU, UK; 3 Department of Psychiatry, Southern Medical University Nanfang Hospital, Guangdong 510515, China; a https://orcid.org/0000-0002-4601-8834

**Keywords:** cancer recurrence, fear, worry, illness representation

## Abstract

There is minimal qualitative research on fear of cancer recurrence (FCR) in patients who are still undergoing treatment. This study explored how breast cancer patients’ illness beliefs changed during radiotherapy treatment, so as to provide their longitudinal perspective across sessions. These beliefs were mapped to Lee-Jones *et al* FCR model to assess its applicability to patients during this key treatment phase. A framework qualitative analysis was employed for verbatim interactions between patients (*n* = 8) and their radiographer (*n* = 2) over a minimum of three weekly review sessions (26 review consultations in total). Results proved suggested evolution and repetition of themes within and across sessions. Most themes were consistent with the early stages of the Lee-Jones et al model (antecedents and FCR) such as internal and external cues, cognitions and emotions. The crucial observation was that somatic stimuli were interpreted as side effects of radiotherapy treatment rather than cancer symptoms. Patients were still undergoing their last phase of major treatment, whereas the Lee-Jones *et al* model has been constructed to explain patients’ past treatment experience. New themes emerged, including current exercise, concurrent illnesses/problems, cancer treatment as a constant reminder (of diagnosis) and associated sleeping difficulties. Decatastrophising of symptoms and experiences relating to cancer and its treatment was also a prominent theme indicating a possible coping mechanism to reduce worries about treatment side effects and associated experiences. Finally, some evidence was found from failure of emotional/fear processing in patients due to early surface reassurance by health professionals – a possible explanation of how FCR might arise. Early detection of FCR and promoting support while patients are still undergoing treatment might prevent patients from developing FCR after treatment.

## Introduction

**Figure 1.**
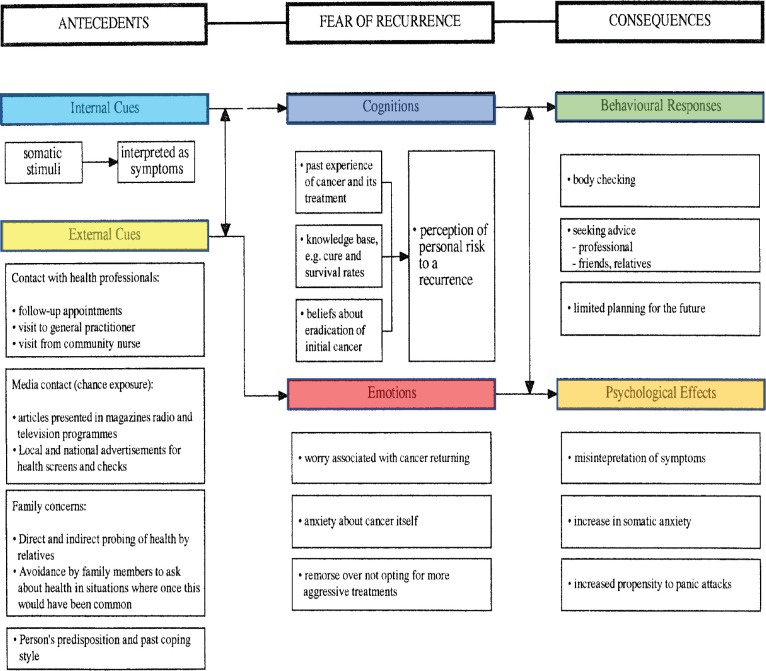
Lee-Jones et al model applied to cancer and fear of recurrence.

Breast cancer is the most common type of cancer in the UK, and it is estimated that one in eight women will be diagnosed with breast cancer in their lifetime. Over half of breast cancer patients (63%) receive radiotherapy as primary cancer treatment [1]. However, after treatment, many people are afraid they still have cancer or that it might return [2]. Leventhal *et al*’ [5] self-regulatory model is one of the main models that explore how people psychologically manage their illness [5], which has been applied to cancer and fear of recurrence (Figure 1). Other approaches exist to assist the explanation of the development of FCR [6, 7]. Our group, which developed the original Lee-Jones *et al* model, has concentrated efforts to develop this model [3].

Leventhal’s self-regulatory model of illness behaviour provides a problem-solving perspective of illness behaviour in which the individual will try to reverse to their normal state prior to illness. The model explores the relationships between symptom interpretation, illness representation and cognition (identity, cause, consequences, timeline and curability/control), emotional response (fear, anxiety and depression), coping (approach and avoidance) and appraisal. A major finding is that the emotional responses experienced by individuals can exacerbate negative symptom interpretation [8, 9].

Furthermore, common-sense beliefs about breast cancer have been investigated post-treatment revealing how perceived severe consequences of cancer lead to changes in lifestyle such as an increase in vegetable consumption and physical activity [2].

The Lee-Jones *et al* FCR model is based on Leventhal’s self-regulatory model of illness behaviour and thus incorporates many similar features [10, 11]. However, the model is specifically applied to cancer with a focus on the fear of recurrence and incorporates the interaction between internal cues (symptom representation) and external cues (health professionals, media, family and the individual’s past coping behaviour). These triggers also interact with cognitions and emotions where the individual’s own perception of risk of recurrence, influenced by their beliefs and negative feelings such as anxiety, play a potential role in FCR development. The consequences of these interactions can lead to both behavioural responses, such as excessive body checking, and negative psychological effects such as increased anxiety and interpreting neutral somatic stimuli as symptoms of cancer. For example, as shown by Soriano *et al* [12], FCR in patients with breast cancer was associated with same-day checking behaviour, triggered by internal and external daily life events, such as skin irritation, tingling sensations and negative interactions with health professionals. These triggers may also be present in patients who are still undergoing treatment rather than only in survivors. As with Leventhal’s model, Lee Jones *et al* model should be interpreted as being dynamic, with feedback loops between cognitions and emotions, as they may fluctuate throughout the treatment triggered by internal and external cues, leading to psychological effects and behavioural responses [13].

A limitation in previous research to examine cognitions, beliefs and associated emotions is the use of direct quantitative measures through self-report questionnaires [14]. Our approach to explore, adequately, individuals’ illness beliefs and behaviours, as well as their impact on emotions, psychological and behavioural responses without creating bias [15] or promoting individuals to develop these representations through assessment, is to use less invasive methods such as observation of natural speech in clinical settings.

The literature shows little qualitative analysis research regarding the application of these models of illness beliefs and behaviour and their application to cancer patients *who are still undergoing treatment.* One important example is the recent report of 12 patients and the detailed analysis of interview transcripts of patients recovering from breast cancer [16]. Interestingly, the model was endorsed with important modifications suggested, especially on the need for support following treatment. The study did not refer to the treatment phase, other than stating that their sample did not express any regret for not ‘opting for more aggressive treatments’, and was based on a single interview with each patient. This Canadian group raise an interesting point that the original Lee-Jones *et al* model concentrated principally on content rather than process. They interpret both their data and cite other FCR models that adopt a metacognitive formulation or a close combination of cognitive and emotional entities to make their important supposition. In response, we consider analysing longitudinal qualitative material. This approach might describe the natural unfolding process of patients’ development of illness representations indirectly [17]. Analysing repeated sessions of patients—longitudinally—interacting with their radiographer, without structured interviews, removes the effect of measurement reactivity in patients’ emotions and cognitions. Our approach may reveal some of the processes in the development of FCR.

We have reported previously on 12 patients from the FORECAST study that completed daily diary ratings of FCR [18]. Selection of two types of patients was conducted, namely, those (*n* = 6) patients with a clear positive trajectory, i.e. increasing FCR, and those (*n* = 6) with a decreasing or negative trajectory. The patients’ content of their emotional conversation was inspected and some evidence of the Lee-Jones *et al* model application was found; however, the methodology was not intended explicitly to systematically map the model onto the acquired data. This current study was focused on directly mapping the Lee-Jones *et al* model onto a new set of patient consultations from the same data corpus of approximately 200 consultations. We focused attention on those patients where we had three or more review appointments over the course of treatment available for analysis. Our research question was to determine if there was evidence to show some of the psychological *processes* outlined in the original Lee-Jones *et al* model in the final treatment phase (i.e. radiotherapy). In other words, could the FCR formulation be reflected in the early period of adjustment for patients following their diagnosis and when primary treatment was about to be concluded, as in many cases, by radiotherapy intervention?

Breast cancer diagnosis, surgery, chemotherapy and radiotherapy treatment produce side effects that can be interpreted as symptoms. These side effects, such as swollen and irritated skin and tingling sensations, can be internal triggers, and promote concern in patients [19]. However, external triggers such as positive and negative contact with health professionals can be an issue with patients undergoing radiotherapy that might also increase self-reported patient FCR [12]. In consequence, the way in which symptoms are perceived can influence illness representations and FCR. Patient beliefs about cancer symptoms and radiotherapy treatment side effects can pose difficulty in interpreting symptoms and further identifying the cause of illness. Hence, to concentrate close attention to this early phase in the patient’s experience of cancer therapy was, to our group, an ideal opportunity to inspect patient psychological experience. The flexibility of qualitative framework analysis will enable possible modification of Lee-Jones *et al* model to breast cancer patients undergoing treatment [20].

### Aims

The aims were to:

- explore how the illness beliefs of patients with breast cancer change longitudinally during radiotherapy, and
- map these beliefs, emotions and behaviours to assess the applicability of the Lee-Jones *et al* fear of recurrence formulation for patients during their final major stage of primary treatment.

## Methods

### Participants and design

A total of 202 consecutive patients, following the computed tomography (CT) scan, were approached; the research study was explained to them by the research assistant (Yuan Yang) with a support from the therapy radiotherapist (Josie Cameron) and they were handed a patient information sheet (PIS). Written consent was given, with 93 refusals (see COREQ form available in Supplementary Data). Of those who proceeded to complete the baseline demographics, the participants available in the final data set (97), a subset of eight breast cancer patients (all women) were sampled. Criteria for entry into this study were: (i) that the participants (from the FORECAST study) had to have a minimum of three recordings of their weekly review session with their therapeutic radiographer, to show the content development of each session, providing a longitudinal perspective, (ii) volunteered, (iii) English spoken, and (iv) no known psychiatric condition. Patients received either a booster treatment or radiotherapy for ductal carcinoma in situ (DCIS). There were no patients with regular radiotherapy treatment (of 15 routine sessions). All patients completed a short seven-item measure of fear of cancer recurrence seven item scale (FCR7) [21] in treatment every week to plot raw scores and assess the variability of FCR over this treatment phase. The FCR7 has good psychometric qualities having been developed with patients with breast, colorectal and head and neck cancer. Scores range from 6 (minimum) to 40 (maximum). The reported internal consistency of the measure was 0.92 (95% CI: 0.90, 0.94) and has evidence for validity. The measure was completed by the patient on a prompt from the research assistant (Yuan Yang) separately from the review sessions.

As applied in our previous work, we used a ‘concurrent mixed-methods explorative design’ [22]. This consisted the FCR rating data from patient self-reports and the transcripts derived from the audio files of radiographer–patient interactions in the review appointments.

### Data analysis

The data collected were analysed principally by descriptive methods. However, some quantitative (regression) and explorative (diagrammatic panel plots) data analyses were performed to understand in detail the variation of the key variable FCR over the course of radiotherapy for this selected group of patients [23]. A generalised linear model (Gaussian distribution) using a robust maximum likelihood estimator was applied to the four ratings of FCR of each patient with week (first, second, third and fourth) as a between-subjects’ factor, controlling for age, living alone or with others, and type of radiotherapy (DCIS or Booster). Statistics were calculated using STATAv15 [24]. As the study was not strictly powered, we refrained from setting an alpha level. We expected some factors to explain the variability of FCR such as patient age which has been shown previously to have a consistent negative relationship [4].

All audiotapes (recorded on stereo recorders: Tascam DR-40 Digital Portable Recorder with SD media) were transcribed verbatim, and a qualitative framework analysis based on Lee-Jones *et al* model of FCR was conducted. Framework analysis has increasingly been used when analysing data in the context of multidisciplinary health research, due to its systematic approach and its ability to summarise complex data [20]. It involves the transformation of qualitative data into codes, allowing comparing and contrasting codes within individual patients, between patients, within and across sessions. Specifically, for this study, all transcripts were coded, and a working framework was developed. A combination of deductive and inductive approach was used to select themes and codes to explore how illness beliefs change during cancer treatment [25]. A deductive approach using codes and themes from Lee-Jones *et al* model was chosen to test the applicability of the stages in Lee-Jones *et al* model regarding fear of cancer recurrence (FCR) and illness behaviour [20, 26]. A framework based on Lee-Jones *et al* FCR model was developed, following these suggested steps, [20] including transcription, familiarisation with data, coding, developing a working analytical framework and applying the framework, and charting data into the framework matrix. The codes were based on the labels of the Lee-Jones *et al* model; however, new codes or slight modifications derived from the content of the sessions were introduced, following an inductive approach, which is generating new codes from the qualitative data that were not present in the original Lee-Jones *et al* model. This additional approach was intended to reflect more widely the patients’ experience while undergoing radiotherapy treatment, without the possible constraint on adhering completely to the original model of Lee-Jones *et al*. The final framework of codes was discussed in detail with Gerry Humphris (the Principal Investigator) who was the originator (with others) of the Lee-Jones *et al* model. We declare that our knowledge of this model would have influenced the recognition of any new codes and this openness and reflexivity would improve the trustworthiness of the final framework constructed [27, 28]. Saturation was not a requirement for this study adopting a framework approach. On completion of three meetings to discuss and finally confirm the new codes, changes to the framework were incorporated and concluded. The codes were assigned using this coding scheme by the lead author (Isabel Del Mar Hita Millan, a health psychology trainee under supervision) and a third of the sample approximately was recoded by Gerry Humphris to assess percentage agreement and calculation of the kappa coefficient [29].

### Ethics

The University of St Andrews and the NHS East of Scotland Research Ethics Committee (NHS Lothian) approved the study (NRES No.: 13/ES/0015). The study was registered with ClinicalTrials.gov (ID No.: NCT02599506).

## Results

**Figure 2.**
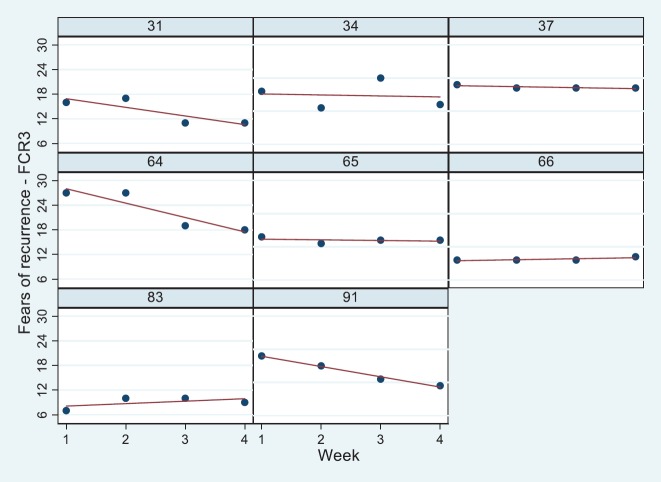
Panel plots for patients 31–91 of their FCR7 scores from start of radiotherapy treatment (week 1) to end (week 4). Linear regression line (red) overlaid.

Of the eight patients sampled, five received radiotherapy for DCIS, whereas three patients received a booster treatment. An equal number of patients were living alone or with others. The mean age of patients was 59 years (SD = 13.2), with a minimum of 39 and a maximum of 75 years. Results of the general linear model indicated that the FCR of eight patients decreased from week 1 to week 4, although not significantly (Table 1). Moreover, those who lived alone and were older reported less FCR levels, whereas those patients who received more extensive treatment (Booster) were more fearful compared to patients with DCIS. The levels of FCR are presented diagrammatically with linear regression lines fitted (Figure 2). Values of all variables are stored in the supplementary file.

From the eight patients, six had three available sessions recorded and two had four sessions recorded. The attendance varied according to the availability of staff and the patient’s schedule. A total of 26 separate sessional transcripts were available for qualitative analysis. Charting of the data (283 identified codes) was conducted using Excel (single code assigned per row), including patient number, session, code and code frequency. In total, seven main features were identified: internal cues (somatic stimuli), external cues, emotions, cognitions, behavioural responses, effects on sleep and current exercise (Table 2). The first three patients’ selected quotes were recoded (*n* = 104) by Gerry Humphris blind using the framework in Table 2. The agreement for the seven main themes was 93% (expected 22%), Kappa = 0.91; SE = 0.048; *z* = 19.4 *p* < 0.0001, and for the full scheme including sub-themes was 86% (expected 9%), Kappa = 0.84; SE = 0.029; *z* = 29.3, *p* < 0.0001.

**Figure 3.**
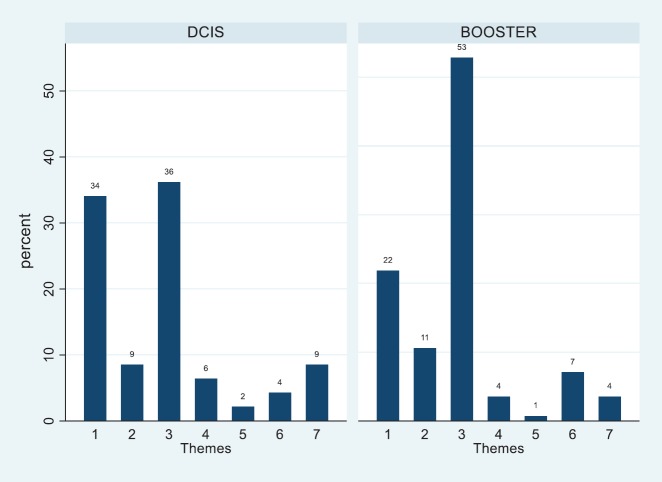
Percentage frequency bar chart of themes 1–7 listed in table 2 across treatment group.

The frequency of codes for each theme by patients was plotted using percentage frequencies, and the plot of the total sample was included for comparison purposes (see supplementary figure). The aggregate percentage frequencies across treatment groups showed significant variation (χ^2^ = 14.58, df = 6, *p* = 0.024). The bar chart (Figure 3) shows that 53% of coded utterances were of an emotional nature in the Booster Group compared with 36% of the DCIS group (overall = 45%). Whereas the DCIS patients raised issues of Internal Cues (34%) more frequently than the Booster counterparts (22%). The overall percentage was 28% for the whole sample. More generally, some of the themes found were similar to those found in breast cancer patients assessed 5 years after the treatment, such as chronic medical problems, acute and transient health problems (concurrent illnesses), life events (concurrent problems) and personal support [30]. This was consistent with the proposal that patients’ retrospective experiences and beliefs match their concerns while undergoing radiotherapy. As patients are undergoing treatment, it is expected that most of the codes would relate to the antecedents of FCR of Lee-Jones *et al* model. An extract of patients’ quotes relating to each code can be seen in Appendix A.

Our ‘Results’ section continues by presenting a selection of the main features of each patients’ psychological and behavioural description of their experience of radiotherapy, with specific reference to the FCR model under study. To assist the reader, a diagrammatic representation of linked constructs is drawn with colour shading derived from the original framework. Linkages are demonstrated using arrows as indicated in the figure legend.

**Figure 4.**
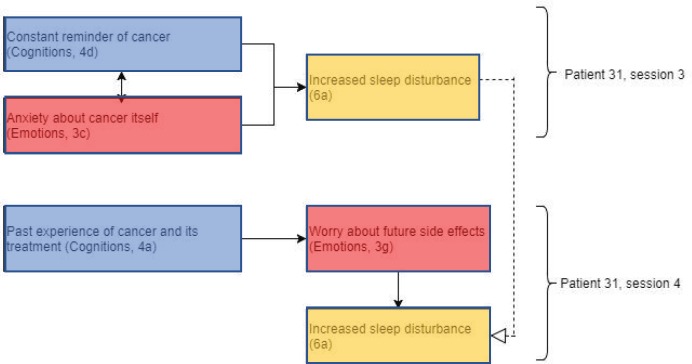
Diagram showing codes for patient 31 in sessions 3 and 4. Black arrows showing relationships within sessions and dashed white arrows showing relationships between sessions. Open bracket cluster constructs with a specified session. Note these presentational conventions are applied with the remaining figures.

Patient 31 showed evidence of repetition of themes within and between sessions, in addition to evidence supporting Lee-Jones *et al* model of FCR (Figure 4). This patient experienced increased sleep disturbance, a theme that was repeated across sessions 3 and 4. This may have been due to an interaction between overthinking of cancer as a constant reminder (cognitions) and anxiety about cancer itself (emotions).

‘I think it’s just, all these things in my mind, what I’m gonna do... I think it was so much thinking about it at night-time’.

This supports the Lee-Jones *et al* model as cognitions interact with emotions, which in turn result in psychological effects. In the case of patients who are undergoing radiotherapy treatment, the psychological effects observed are related to sleep disturbance rather than symptom misinterpretation or panic attacks. Patient 31 revealed that she was a breast cancer survivor and underwent radiotherapy treatment 12 years ago before her current treatment. This may explain why the theme of past experience of cancer and its treatment (cognitions) was discussed in depth in sessions 1, 3 and 4, as the patient reminisces about her own experience, using these cognitions to predict what to expect from receiving the same treatment again. Relying on these past experiences and being familiar with the expected side effects of radiotherapy, it is unsurprising that the patient would worry about future side effects, especially if her previous experience had been troubling.

Patient 91 discussed similar themes to patient 31, such as overthinking and cancer as a constant reminder (cognitions) and contributed to worry about future side effects and increased sleep disturbance. However, in addition to this, the patient also indicated worry about the current side effects of radiotherapy treatment and somatic stimuli attributed to radiotherapy treatment and concurrent illnesses.

‘Sometimes I get pins and needles feelings like that’.

Somatic stimuli such as these can act as triggers for the patient to think about cancer and result in overthinking, which in turn may increase sleep disturbance.

‘I think [31] is not switching off’.

Unlike patient 31, patient 91 displayed moderate fear of recurrence (total FCR7 score of 18), perhaps explained by the addition of concurrent illnesses, increasing somatic stimuli (internal cues) that may act as triggers of fear of recurrence.

**Figure 5.**
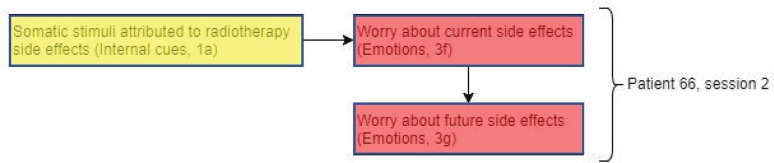
Diagram showing codes for patient 66 in session 2.

Interestingly, patient 66 (Figure 5) shows a similar pattern to patient 91. However, instead of worry about current side effects repeating across sessions, the phenomenon was observed within the same session. Similar to patient 91, these worries could have been triggered by somatic stimuli attributed to side effects of radiotherapy.

‘[This Breast is] a tiny bit weird compared to the other side [...] I’ve got a rash that started and it’s irritating’.

Again, this interaction between internal cues (somatic stimuli) triggering worries supports the Lee-Jones *et al* model. Even though patient 66 shows a similar pattern to patient 91, fear of recurrence scores remained low at baseline and throughout treatment. This may be due to the lack of sleep disturbance and, therefore, less opportunity of lying awake and having cancer as a constant reminder and overthinking. A possible explanation of how fears of recurrence might arise is by a failure of emotional processing [40]. In an attempt by the radiographer to reassure patient 66 about the side effects of the radiotherapy treatment, this may block the patient from processing the fears.

Patient 66: ‘It started off a bit as a sore throat but now I’ve got a tickly throat’.

Radiographer: ‘We’re not treating as high as your throat though so’.

Patient 66: ‘No, I know but I was just feeling this, it’s going to make me cough’.

Radiographer: ‘Aha yeah. It’s definitely not radiotherapy related but em, any other questions?’

This rapid change of subject eradicates the opportunity to talk about possible radiotherapy side effects which could promote emotional processing. The radiographer’s attempt to reassure the patient appeared not to be successful as this way further mentioned in session 2, with the patient expressing uncertainty about the cause of illness.

‘And nobody else I know has a cold. I don’t know where I got it from’.

Perhaps, allowing the patient to express their concerns about side effects rather than interrupting the patient’s description of symptoms could not only promote emotional processing but also reduce the fear of cancer progression [41].

**Figure 6.**
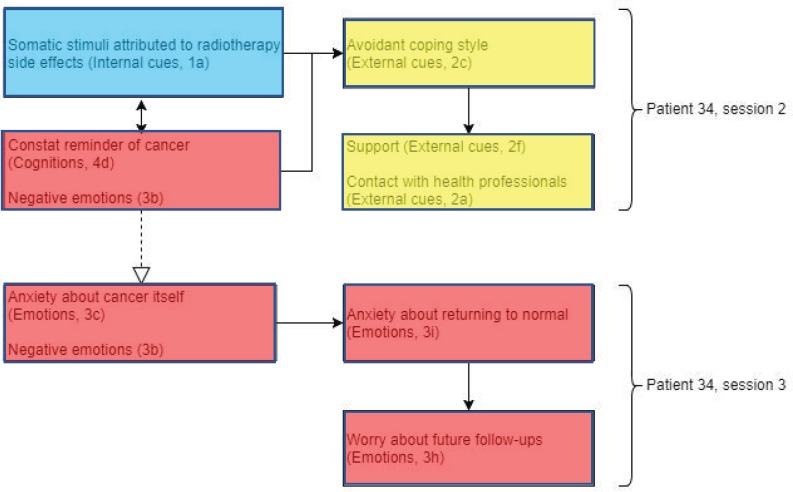
Diagram showing codes for patient 34 in sessions 2 and 3. Black arrows showing relationships within sessions and dashed white arrows showing relationships between sessions.

Patient 34 (Figure 6) displayed somatic stimuli attributed to the side effects of radiotherapy, accompanied by tiredness. Experiencing side effects of radiotherapy, such as tingling or tender skin, can also trigger the patient to be frequently reminded about their diagnosis of cancer. In addition to this, very negative emotions associated with uncertainty and low mood were expressed.

‘like sunburn or something, it’s just there [...]’it’s in your head as well’

‘I don’t know… I’m just low’.

This low mood resulted in the patient’s avoidant coping style using alcohol as a way of dealing with having to visit the hospital every day for radiotherapy treatment. However, this prompted the patient to converse with her family and the option of attending group therapy as a support to reduce her low mood. This supports the Lee-Jones *et al* model as there is evidence of how external cues (support from family and health professionals) may influence the person’s emotions and coping styles. Positive emotions were observed in session 3, suggesting relief as the patient approached the end of radiotherapy treatment. Therefore, the impression exhibited from this patient was mixed; however, the negative emotions and cancer as a constant reminder from the previous session possibly conflicted with these positive emotions to maintain the anxiety about cancer itself.

‘Scary in the first place for somebody to say you’ve got cancer’.

Cancer as a constant reminder and avoidant coping style in addition to anxiety about cancer itself may explain the patient’s moderate fear of recurrence, supporting the evidence of external cues and emotions present in the Lee-Jones *et al* model. The patient’s realisation of radiotherapy treatment coming to an end may indicate some evidence of her anxiety returning to a normal state.

‘What am I supposed to do with myself? Just to think now that it’s finishing’.

This, in turn, prompted the patient to ask about the invasiveness of future follow-up tests after finishing radiotherapy treatment.

Interestingly, patient 64 also displayed very similar themes to patient 34, such as somatic stimuli attributed to radiotherapy side effects, negative emotions and cancer as a constant reminder. Negative emotions were associated with worsened side effects produced by radiotherapy treatment and surgery.

‘If I get agitated, [19] gets sore’.

This interaction between emotions and somatic stimuli (internal cues) supports the Lee-Jones *et al* model, showing a circulatory feedback loop between these elements. In addition, somatic stimuli such as tingling sensations and soreness may trigger emotions relating to cancer, and cancer as a constant reminder (cognitions).

‘I don’t think you want to dwell on it really either, because it’s not helping you, sometimes you’re not really thinking about it but it’s here’.

The combination of negative emotions and cancer as a constant reminder triggered by somatic stimuli could explain that the patient’s 64 total FCR scores at baseline and throughout the treatment are high.

**Figure 7.**
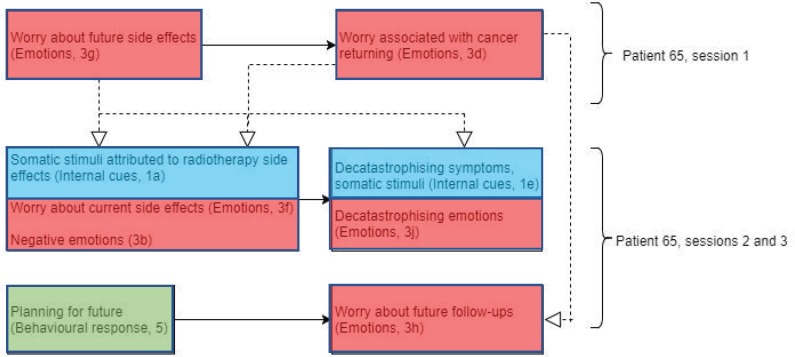
Diagram showing codes for patient 65 in sessions 1–3.

Patient 65 (Figure 7) displayed worries about future side effects and what to expect early in session 1. This, in turn, resulted in the patient explicitly addressing FCR due to opting not to take chemotherapy. This might have been triggered by worries of future side effects.

‘Is there anything...to look out for?’

‘You’ve got to make the decision that even...if you’ve been taken it, it might recur’.

This worry of cancer returning in session 1 may be linked to worries about future follow-ups in session 3, which is also triggered by the patient’s planning of future holidays. In addition to this, worries about current side effects and somatic stimuli attributed to side effects of radiotherapy treatment were discussed in sessions 2 and 3. This may have been due to the fact that as radiotherapy treatment advances, side effects accumulate, meaning that patients may experience more noticeable and uncomfortable side effects.

‘I felt really rough and I felt quite nauseous, just really off and I thought, ooh, this is the start of it’.

Interestingly, patient 65 decatastrophised both physical symptoms and emotions in both sessions 2 and 3. This suggests that decatastrophising of symptoms and emotions (experiences about the treatment) may be a form of coping style, in order to reduce these negative emotions.

‘It’s a lot less pink than I thought it was going to be’

‘It’s not the worst’.

If decatastrophising symptoms and emotions regarding cancer and radiotherapy treatment could be considered a form of coping style, this supports the Lee-Jones *et al* model, showing an interaction between somatic stimuli (internal cues), external cues (coping style) and emotions.

Patient 83 also shows evidence of decatastrophising symptoms triggered by somatic stimuli attributed to side effects of radiotherapy in sessions 1 and 2. In session 3, worries about current side effects were expressed, which resulted in decatastrophising emotions regarding radiotherapy treatment. Interestingly, this patient used the same indicator of skin colour to estimate side effect severity:

‘That’s pink, I mean it’s not anything terrible’

‘the travelling [to hospital] ...it’s not a *big deal *but you get fed up with it’

This would further support decatastrophising as a form of coping style to reduce negative emotions and worries regarding somatic stimuli. A low FCR level throughout the treatment suggested that decatastrophising might be an appropriate coping style for some patients.

Patient 37 displays similar themes to patient 65, such as somatic stimuli, negative emotions, worry about future side effects, worry about future follow-ups, future planning and explicit worry associated with cancer returning.

‘Can you be quite confident that, you know, that it [cancer] won’t come back?’

Patient 37’s fear of recurrence mentioned in session 2 could have been triggered by somatic stimuli attributed to the side effects of radiotherapy treatment (internal cues), which in turn prompted worries about current side effects and worries about future follow-ups in session 4.

Overall, four out of the eight patients showed evidence of planning for the future, such as holidays (behavioural response). Some of the patients who are still undergoing radiotherapy treatment plan holidays as a future reward for finishing treatment or as a break between treatment sessions. Long term follow-up of these patients would predict that their FCR levels will be less according to the Lee-Jones *et al* model. In addition, another prominent theme mentioned by every single patient who does not appear in Lee-Jones *et al* model was ‘exercise’. An explanation for this is that radiographers recommend patients who are still undergoing treatment to carry out a light exercise to alleviate radiotherapy-related fatigue [32]. Again the recording of levels of physical exercise, both retrospective and post-treatment, would be important to associate with FCR development. A protocol of a new RCT to test the benefits of a physical exercise motivator through telephone counselling, on energy levels and FCR has been reported and will be an important test of the potential benefits of this lifestyle behaviour as well as of the intervention technique [33].

## Discussion

A mixed-methods approach was applied to a data corpus obtained from eight patients who were in discussion with their therapeutic radiographer over a minimum of three occasions during their radiotherapy treatment. The quantitative findings showed a clear association of FCR with patient age as predicted in [34]. In addition, patients with more extensive disease reported higher FCR levels compared to patients with early signs of malignancy (DCIS). On average, the trajectory over the course of treatment was relatively stable in this group of eight patients, although there appeared to be a trend to lower FCR as treatment progressed reflecting our previous findings elsewhere [35]. Inspection of the raw data in the panel plots showed, in addition, some variation.

Examining perceptions, cognitions and emotional content from utterances between breast cancer patients and their radiographers across sessions provided a unique longitudinal perspective. The level of agreement in the coding of the patient utterances was very good and enables some trustworthiness in the accurate identification of the elements of the Lee-Jones *et al* model. There was partial evidence supporting Lee-Jones *et al* model of fear of recurrence in patients with breast cancer who were undergoing radiotherapy treatment. This is the first attempt to observe vicariously the development of expressed patient health beliefs and emotions from staff and patient interactions during radiotherapy treatment intervention. Of the codes assigned, as defined from the framework, it was found that they supported the elements clustered in the antecedents stage (internal and external cues as triggers) and some of the elements of fear of recurrence stage (cognitions and emotions) of the Lee-Jones *et al* model. There was evidence supporting contact with health professionals, media contact, predisposition, past coping style and family concerns (external cues) as triggers for negative emotions and worries as seen in Lee-Jones *et al* model. In addition, there was a code assigned to support all cognition elements in this model, namely, past experience of cancer and its treatment, knowledge base and beliefs about the eradication of initial cancer, all mediating the perception of personal risk to a recurrence [36]. Moreover, two other cognitive processes were identified: ‘cancer as a constant reminder’ and ‘overthinking’, often triggered by somatic stimuli attributed to side effects of radiotherapy treatment. These were identified in half of the patients.

There was evidence to support some of the emotions found in Lee-Jones *et al* model, such as worry associated with cancer returning and anxiety about cancer itself, whereas worry was associated with: (i) treatment, (ii) current side effects, (iii) future side effects, (iv) follow-ups and (v) returning to normal. However, during the radiotherapy treatment phase, notably, we did not find any evidence of patients attributing internal cues (somatic stimuli) to symptoms of cancer. This was unsurprising as the Lee-Jones *et al* model is applied to patients who have already finished treatment, whereas the current framework analysis was applied to patients who are undergoing radiotherapy treatment. Therefore, it was expected that most of the somatic stimuli would be interpreted as side effects of radiotherapy. Likewise, the majority of emotions patients presented involved worry about treatment and its side effects. In the context of the interactions of the patients with their radiographer, there was no evidence that the aforementioned worries (i)–(v) were associated with expressed anxieties about cancer itself, thereby matching the finding of Maheu *et al* [16] in their qualitative work.

The theme of ‘current exercise’ was present in all patients still undergoing radiotherapy treatment, which was not shown in Lee-Jones *et al* model (for patients who have finished treatment). However, the theme: ‘current exercise’ was difficult to categorise; in some cases this was negative, as it resulted in an increase of somatic stimuli and tiredness or was associated with negative emotions, such as frustration (as patients were not able to engage the same level of exercise as they previously could before treatment). In other cases, exercise was a positive element, helping reduce radiotherapy related fatigue. In addition, some patients mentioned exercise as a form of coping. In this case, exercise as a form of coping style (external cues) would be appropriately considered as part of the Lee-Jones *et al* model.

‘[exercise] helps to take your mind off of feeling sorry for yourself.’

There was no patient who described exercise as a means to reduce cancer recurrence risk. This was not surprising as the expanded self-regulation model presented by Durazo and Cameron [37], which presented mixed evidence of the link between physical exercise and recurrence worry, was specified for patients following their treatment. Similarly, decatastrophising symptoms and emotions regarding radiotherapy treatment are not integrated with the Lee-Jones *et al* model. They were present in seven out of our eight patients who were still undergoing radiotherapy treatment. The patients who expressed anxiety about the treatment may be voicing a natural response to an uncertain and novel experience. However, if decatastrophising symptoms were to be interpreted as a form of coping style, this would be explained by the Lee-Jones *et al* model. It is unclear whether decatastrophising symptoms would reduce FCR, although this would be predicted by Leventhal’s wider self-regulation formulation that the Lee-Jones *et al* model was drawn from [8].

A theme that was present in patients undergoing radiotherapy treatment that does not fit the current Lee-Jones *et al* model is ‘concurrent problems’, which may be classified as internal cues due to other co-morbid illnesses or external cues due to other problems, such as life events. This theme was present in seven out of eight patients and supports Mills *et al*’s [30] findings of breast cancer survivors considering chronic medical problems, acute health problems and life events as concerns when they underwent treatment. In addition, staff dismissing patients’ symptoms and attributing them to concurrent illnesses rather than radiotherapy treatment side effects may impede emotional processing rather than reassure patients about their symptoms. This could be linked to the development of FCR. To avoid this process, a potential remedy would be for health professionals to allow their patients to fully describe their symptoms and concerns rather than dismiss them rapidly as unrelated to radiotherapy treatment.

In summary, the data exhibit a varied pattern of how breast cancer patients’ illness beliefs changed longitudinally during radiotherapy treatment, with repetition of themes within, between sessions and between patients. This pattern may, of course, be explained by the varied engagement level of the radiotherapy staff member; however, overall, somatic stimuli (internal cues) attributed to side effects of radiotherapy treatment and negative emotions were the most prominent themes, often mentioned by patients in the same set of utterances within a single review session.

### Limitations and future directions

This report was explorative and not designed to be powered statistically for a specific effect size or to reach saturation. In addition, the researchers who analysed the verbatim transcripts recognised that they reviewed the material from a particular framework that had been developed from this research group, namely, a parallel-process cognitive-emotional model. The results presented need to be interpreted from this perspective. It is acknowledged that the material presented can be understood from multiple formulations; however, the model adopted, we believe, has a degree of faithfulness to provide some compelling evidence to reveal some important processes in these patients which have yet to be explicitly revealed.

These data referred to female patients with breast cancer from a single specialist treatment unit. There is no assumption of generalisability across other cancers or gender. Future research should extend the use of recorded sessions between patients and radiographers to replicate and confirm the applicability of the Lee-Jones *et al* model. We note the work of Maheu *et al* [16] who have used qualitative methods to suggest some changes to the Lee-Jones *et al* model. Their suggestion to conflate cognitions and emotions as they are so integrated with the patient experience is interesting and faithfully reflects patient description when interviewed. We suggest that theoretically however some separation is warranted to enable researchers, and clinicians who professionally support these patients, to possess a working framework to develop a closer interpretation of the possible links between cognition and emotion. This makes explicit a genuine parallel process model (see Love *et al* [38], for example, in the chemotherapy setting) that can be recognised by staff when working with patients on a daily basis in their cancer service, and employed for potential therapeutic purposes. We believe that close inspection using intensive longitudinal methodology will enable a teasing out of these processes to the advantage of our understanding of a crucial concern for the majority of patients with cancer [39].

## Conclusion

This attempt to observe patients with breast cancer and infer their cognitions and emotions from verbatim discussions with their radiographer when they are still undergoing radiotherapy treatment shows evolving themes throughout treatment, with some repetition within and between sessions. There is evidence to support elements of the Lee-Jones *et al* model, especially related to antecedents and fear of recurrence stages, such as internal and external cues, cognitions and emotions. The possibility exists that staff working with these patients through an understanding of this model and its processes may be able to develop ways to prevent extensive fears of cancer recurrence developing into the immediate post-treatment phase of recovery.

## Availability of data and materials

Requests to the corresponding author.

## List of abbreviations

IPQ: Illness perception questionnaire.
SRQ: Symptom representation questionnaire.

## Conflicts of interests

The authors declare that they have no conflicts of interest.

## Funding

Generous support received from Breast Cancer Now (Grant No. 6873). This funding body was independent of study design, data collection, interpretation and manuscript writing.

## Authors contributions

Isabel Del Mar Hita Millan transcribed the recorded patient–therapy radiotherapist interactions, analysed the qualitative data, prepared the first draft of the manuscript and reviewed all versions subsequently. Yuan Yang participated in consenting patients, collected, entered and checked all data, and in manuscript preparation. Josie Cameron assisted consenting, study design and manuscript review. Gerry Humphris participated in the design of the study, measure construction, detailed statistical analysis and manuscript preparation. All authors read and approved the final manuscript.

## Figures and Tables

**Table 1.** General linear model with fear of recurrence (FCR7) regressed on four factors using robust estimator.

	B	SE	*z*	*p*	95% CI	
**Factor**					**Lower**	**Higher**
**Alone**^¥^	−4.13	1.54	−2.69	**0.007**	−7.14	−1.12
**Boost treatment**^§^	7.65	2.28	3.36	**0.001**	3.19	12.11
**Week versus 1st**						
** 2nd**	−0.88	2.44	−0.36	0.720	−5.66	3.91
** 3rd**	−1.88	2.34	−0.80	0.423	−6.46	2.71
** Final**	−3.25	2.10	−1.55	0.121	−7.36	0.86
**Age in years**	0.21	0.10	2.03	**0.043**	0.01	0.41
**Constant**	1.92	6.59	0.29	0.770	−10.99	14.83

¥ 1 = lives alone; 0 = lives with others;

§ 1 = Booster radiotherapy; 0 = DCIS radiotherapy treatment

**Table 2.** Coding scheme showing seven main themes and their sub-themes.

No.	Theme	Sub-themes
1.	Internal Cues (Somatic Stimuli)	Attributed to side effects of radiotherapy Attributed to side effects of medication, surgery or healing Attributed to attitude or age Attributed to concurrent illnesses Decatastrophising symptoms
2.	External cues	Contact with health professionals Coping styles: positive negative Predisposition Concurrent problems Support (family, friends, others) Media contact
3.	Emotions	Positive Negative (guilt, uncertainty, frustration, tiredness) Anxiety about cancer itself Worry associated with cancer returning Anxiety about treatment Worry about current side effects Worry about future side effects Worry about future follow-ups Anxiety about returning to normal Decatastrophising symptoms
4.	Cognitions	Past experience of cancer and its treatment Beliefs about eradication of initial cancer Knowledge base Constant reminder of cancer / overthinking
5.	Behavioural responses	Planning for future
6.	Effects on sleep	Increased sleep disturbance Decreased sleep disturbance
7.	Current exercise	

## Consolidated criteria for reporting qualitative studies (COREQ): 32-item checklist

Developed from:

Tong A, Sainsbury P, Craig J. Consolidated criteria for reporting qualitative research (COREQ): a 32-item checklist for interviews and focus groups. *International Journal for Quality in Health Care*. 2007. Volume 19, Number 6: pp. 349 – 357

**Table:**

No. Item	Guide questions/description	Reported on Page #
**Domain 1: Research team and reﬂexivity**		
*Personal Characteristics*		
1. Interviewer/facilitator	Which author/s conducted the interview or focus group? Not applicable	N/A
2. Credentials	What were the researcher’s credentials? E.g. PhD, MD The PI has a PhD, a Master’s in Clinical Psychology, Chartered Psychologist status with the British Psychological Society: BPS Reg #: 09951; and a member of the Health Care Professions Council: Reg #: PLY15944 Millan is a Health Psychologist (MSc), Yuan a psycho-therapist (Msc) and Cameron is a Therapy Radiographer with BSc and Diploma of College of Radiographers	Page 1
3. Occupation	What was their occupation at the time of the study? GH: Chair of Health Psychology and Hon Consultant of Clinical Psychology NHS Lothian; IDMHM: MSc Trainee; YY: MSc Research Assistant; JC: Senior Therapy Radiographer	Page 14
4. Gender	Was the researcher male or female? IDMHM; YY, JC: Female/ GH: Male	Not explicitly stated
5. Experience and training	What experience or training did the researcher have? IDMHM: 1 year trainee; YY: 2 year trainee and research assistant; JC: Practitioner researcher over 5 years +; GH: Health services and health care communication researcher over 40 years + , e.g. Chair of Standing Committee on Research of the International Association of Communication in Healthcare (2014-2018) www.https://www.each.eu/	Not explicitly stated
*Relationship with participants*		
6. Relationship established	Was a relationship established prior to study commencement? Consecutive patients consented by research assistant and radiographer in clinic during time of treatment delivery to participants	Page 12
7. Participant knowledge of the interviewer	What did the participants know about the researcher? e.g. personal goals, reasons for doing the research Full patient information sheet (PIS) passed by NRES NHS Committee that outlined objectives of research	Page 12
8. Interviewer characteristics	What characteristics were reported about the interviewer/facilitator? e.g. Bias, assumptions, reasons and interests in the research topic The data collected were observations (as outlined in the PIS). These were verbatim transcripts of the interaction between radiographer (therapy) and the patient during weekly review meetings. Hence these meetings were routine review consultations without a research agenda. The patient was aware that the consultations were recorded for future analysis of emotional response to treatment and the management of their treatment during their daily visits for radiotherapy. The researcher analyzing the transcripts was a masters student with a particular interest in fear of cancer recurrence (FCR) and extensive knowledge of the Lee-Jones et al FCR Model.	Page 12
**Domain 2: study design**		
*Theoretical framework*		
9. Methodological orientation and Theory	What methodological orientation was stated to underpin the study? e.g. grounded theory, discourse analysis, ethnography, phenomenology, content analysis Essentially this was a content analysis with a well-known framework outlined by the Lee-Jones et al FCR Model	Page 13
*Participant selection*		
10. Sampling	How were participants selected? e.g. purposive, convenience, consecutive, snowball Consecutive and included those patients that had interactions of 3 or 4 weekly taped recordings completed	Page 12
11. Method of approach	How were participants approached? e.g. face-to-face, telephone, mail, email Face-to-face	Page 12
12. Sample size	How many participants were in the study? Total sample of 97 patients of which 8 were included that had full information on 3 to 4 review recordings and FCR questionnaire ratings	Page 12
13. Non-participation	How many people refused to participate or dropped out? Reasons? A total of 202 patients were approached after CT scan and 93 patients refused to participate in the study. The major reasons for refusal were: not wanting to be reminded of cancer (62%); not interested (19%); or too busy (9%). Finally, the total number of breast cancer patients enrolled in the study was 97 (response rate, 48%)	Page 12
*Setting*		
14. Setting of data collection	Where was the data collected? e.g. home, clinic, workplace Clinic	Page 12
15. Presence of non-participants	Was anyone else present besides the participants and researchers? No	Page 12
16. Description of sample	What are the important characteristics of the sample? e.g. demographic data, date All patients who were being treated with breast cancer attending a specialist hospital treatment centre. Demographic data included in paper	Page 14
*Data collection*		
17. Interview guide	Were questions, prompts, guides provided by the authors? Was it pilot tested? Not applicable as the recordings were patient-centred and the content was led by patient concerns following radiographer open ended questions about ‘how the patient was with their treatment?’	Page 13
18. Repeat interviews	Were repeat interviews carried out? If yes, how many? As explained above the patients made repeated visits (from 3 up to 4 consultations separated by a week in all cases)	Page 15
19. Audio/visual recording	Did the research use audio or visual recording to collect the data? Audio-stereo digital recordings	Page 13
20. Field notes	Were field notes made during and/or after the interview or focus group? Questionnaire completion of FCR ratings at each consultation	Page 12
21. Duration	What was the duration of the interviews or focus group? Average duration was approximately 10 minutes	Page 15
22. Data saturation	Was data saturation discussed? Saturation was not applicable to this study as the objective was to determine whether the patient interactions reflected the formulation that the Lee-Jones et al model had outlined in previous work with patients with cancer	Page 14
23. Transcripts returned	Were transcripts returned to participants for comment and/or correction? No, The audio-recordings were collected and stored onto a safe haven for research team to have access alone	Not explicitly stated in paper
**Domain 3: analysis and findings**		
*Data analysis*		
24. Number of data coders	How many data coders coded the data? Two	Page 14
25. Description of the coding tree	Did authors provide a description of the coding tree? Coding based upon Lee-Jones et al model	Page 14
26. Derivation of themes	Were themes identified in advance or derived from the data? Advanced with some room for additional constructs to be added if required	Page 14
27. Software	What software, if applicable, was used to manage the data? Excel spreadsheet	Page 16
28. Participant checking	Did participants provide feedback on the findings? No	Not explicitly stated in paper
*Reporting*		
29. Quotations presented	Were participant quotations presented to illustrate the themes/findings? Was each quotation identified? e.g. participant number Quotations supplied with patient number linked	Page 18 to 27
30. Data and findings consistent	Was there consistency between the data presented and the findings? Researchers make links between the L-J FCR Model and quotations	Page 18 to 27
31. Clarity of major themes	Were major themes clearly presented in the findings? Yes	Page 18 to 27
32. Clarity of minor themes	Is there a description of diverse cases or discussion of minor themes? All cases were described in detail and revealed a mix of description from very detailed consistency to minor themes to an overall less descriptive match to major themes	Discussion of major and minor themes Page 18 to 27
